# Multiple plasma membrane reporters discern LHFPL5 region that blocks trafficking to the plasma membrane

**DOI:** 10.1038/s41598-023-28045-w

**Published:** 2023-02-13

**Authors:** David C. Soler, Angela Ballesteros, Andrew E. Sloan, Thomas S. McCormick, Ruben Stepanyan

**Affiliations:** 1 Department of Neurosurgery, University Hospitals Cleveland Medical Center, Cleveland, OH USA; 2 Brain Tumor and Neuro-Oncology Center, University Hospitals Cleveland Medical Center, Cleveland, OH USA; 3 University Hospitals-Cleveland Medical Center and the Case Comprehensive Cancer Center, Case Western Reserve University School of Medicine, Cleveland, OH USA; 4 National Institute on Deafness and Other Communication Disorders, National Institutes of Health, Bethesda, MD USA; 5 Department of Dermatology, Case Western Reserve University, Cleveland, OH USA; 6 Murdough Family Center for Psoriasis, University Hospitals Case Medical Center, Cleveland, OH USA; 7 Department of Otolaryngology – HNS, Case Western Reserve University, Cleveland, OH USA; 8 Department of Neurosciences, Case Western Reserve University, Cleveland, OH USA

**Keywords:** Biological techniques, Biochemistry, Proteins

## Abstract

The mechano-electrical transduction (MET) channel of the inner ear receptor cells, termed hair cells, is a protein complex that enables our senses of hearing and balance. Hair cell MET requires an elaborate interplay of multiple proteins that form the MET channel. One of the MET complex components is the transmembrane protein LHFPL5, which is required for hair cell MET and hearing. LHFPL5 is thought to form a multi-protein complex with other MET channel proteins, such as PCDH15, TMIE, and TMC1. Despite localizing to the plasma membrane of stereocilia, the mechanosensing organelles of hair cells, LHFPL5 requires its binding partner within the MET complex, PCDH15, to localize to the stereocilia tips in hair cells and to the plasma membrane in heterologous cells. Using the Aquaporin 3-tGFP reporter (AGR) for plasma membrane localization, we found that a region within extracellular loop 1, which interacts with PCDH15, precludes the trafficking of AGR reporter to the plasma membrane in heterologous cell lines. Our results suggest that the presence of protein partners may mask endoplasmic reticulum retention regions or enable the proper folding and trafficking of the MET complex components, to facilitate expression of the MET complex at the stereocilia membrane.

## Introduction

In the inner ear, sensory hair cells use mechano-electrical transduction (MET) to convert mechanical forces into electrical signals, initiating the transmission of auditory and vestibular information. The correct targeting of MET pore forming transmembrane channel-like 1 (TMC1) protein to the tips of shorter stereocilia, the site of MET in hair cells, as well as the proper function of MET channels, rely on an alliance of multiple proteins that assemble the MET channel^1,2^. One of the components of the MET channel complex is Lipoma HMGIC (high mobility group protein I-C, now known as HMGA2) fusion partner-like 5 (LHFPL5). LHFPL5, which was previously known as TMHS (tetraspan membrane protein of hair cell stereocilia), is necessary for proper MET channel function^3^. LHFPL5 is a four transmembrane segment protein from the superfamily of tetraspan proteins that includes the claudin tight junction proteins, gap junction proteins, peripheral myelin proteins, and ion channel auxiliary subunits^4^. Mutations in LHFPL5 may impair auditory and vestibular function in zebrafish, cause congenital hearing loss in mice, and DFNB67 deafness in humans^5–8^.

Peak expression of LHFPL5 in the murine inner ear is between P0 and P3 corresponding with the period of stereocilia formation and MET acquisition^9,10^. LHFPL5 is detected on the apical hair cell membrane and stereocilia of both inner and outer hair cells, and in hair cells of the vestibular maculae and cristae^9,11^. Immunogold labeling revealed that LHFPL5 is present in hair bundles at P0 with the expression level peak at about P3^10^. LHFPL5 localizes to the tips of predominantly shorter stereocilia, the site of MET channels, and transiently to the kinocilium and lower shaft and ankle link areas^10^.

Initially, LHFPL5 was thought to be involved in the formation of hair cell stereocilia and hair bundle morphogenesis due to its spatial and temporal expression pattern. However, co-immunoprecipitation experiments and structural studies found that LHFPL5 interacts with MET channel complex components, protocadherin-15 (PCDH15)^3,12^ and TMIE^13^, pointing to a role in MET for LHFPL5. Interestingly, LHFPL5 fails to localize to the plasma membrane of cultured cells and to the stereocilia tips in the absence PCDH15^3,10^, suggesting that the formation of an LHFPL5-PCDH15 complex is necessary for proper LHFPL5 localization. PCDH15 contains a single transmembrane helix and its intracellular C-terminus interacts with the N-terminus of TMC1^12,14^. Earlier reports demonstrated no evidence for co-immunoprecipitation of LHFPL5 by TMC1^15^, but a more recent study detected an interaction between the two proteins using co-immunoprecipitation experiments in heterologous expression systems^16^. It is possible that LHFPL5 may stabilize the interaction between PCDH15 and TMC1; in the absence of TMC1, PCDH15 and LHFPL5 are still targeted to stereocilia^3^, while pathogenic mutations of PCDH15 or LHFPL5 affect targeting of TMC1 to the stereocilia^15^.

LHFPL5, thus, resembles other ion channel regulatory subunits such as the TARPs of AMPA receptors that facilitate channel transport and regulate the properties of pore-forming channel subunits, but the LHFPL5 function remains unknown^17^.

LHFPL5 expressed in cultured cells consistently fails to localize to the plasma membrane and is retained intracellularly^3^. The failure of LHFPL5 to traffic to the plasma membrane in a heterologous system is a major obstacle to identifying and studying the function of this protein. Interestingly, co-expression with PCDH15 has been shown to allow for plasma membrane expression of both proteins, LHFPL5 and PCDH15^3^, suggesting that intracellular assembly of protein complexes may be indispensable for proper localization and function of these MET channel proteins.

Here, we took advantage of Aquaporin 3-tGFP (AQP3-tGFP) fusion protein, which unambiguously displays intense plasma membrane labeling in heterologous systems, to identify LHFPL5 sequences that prevent the plasma membrane localization of AQP3-tGFP. Using AQP3-tGFP based reporter (AGR)^18,19^, we identified a region within LHFPL5 that precludes its expression at the plasma membrane of HEK293 and CHO cells: the extracellular loop 1 (ECL1). Identifying the regions of LHFPL5 that preclude this protein from localizing to the plasma membrane will aid future studies aimed at reconstitution of the MET complex proteins at the plasma membrane in heterologous cells.

## Results

### AGR reveals amino acid sequence LHFPL5^47–96^ prevents trafficking to the plasma membrane in a heterologous cell line

**Figure 1:**
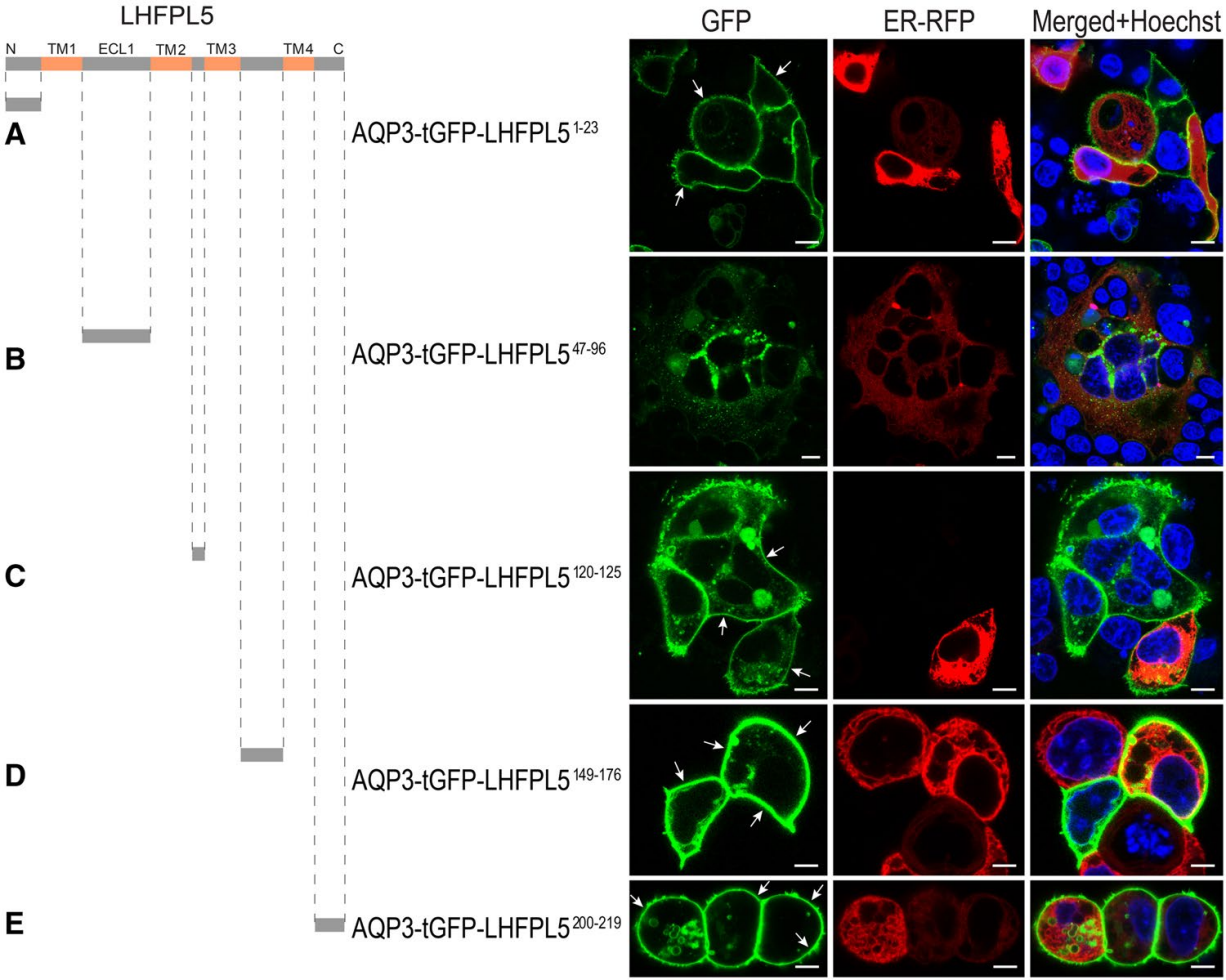
AGR discerns the ability of LHFPL5 regions to halt trafficking to the PM. AGR-based method was used to examine whether any of intracellular and extracellular loops, as well as N- and C-termini of LHFPL5 have the ability to preclude any detectable reporter trafficking to the PM. (**A**) The N-terminus fragment of LHFPL5, comprising residues LHFPL5^1–23^, does not preclude trafficking of the AGR system to the PM. When expressed in HEK293 cells, AQP3-tGFP-LHFPL5^1–23^ exhibit robust PM localization (white arrows). (**B**) In contrast, extracellular loop 1, consisting of LHFPL5^47–96^ fragment, halts any detectable trafficking to the PM. AQP3-tGFP-LHFPL5^1–23^ expression shows an intracellular only labeling pattern. (**C–E**) AGR-based constructs containing the remaining intracellular loop 1, LHFPL5^120–125^ (**C**), extracellular loop 2, LHFPL5^149–176^ (**D**) or the C-terminus, LHFPL5^200–219^ (**E**) show PM labeling. Scale bars: 10 µm. ER was labeled with ER-red fluorescent protein (ER-RFP) in all figures.

The LHFPL5 gene encodes a membrane protein of approximately 24 kDa. The recent cryo-EM study of the LHFPL5 in complex with PCDH15 (PDB ID: 6C14) revealed the LHFPL5 structure^12^. LHFPL5 presents intracellular N- and C-terminal domains and four transmembrane segments resulting in two extracellular loops (ECL1 and ECL2) and one short intracellular loop (ICL). Employing the AGR system, we examined all LHFPL5 extracellular and intracellular loops, as well as both termini, to identify regions that have the ability to preclude protein trafficking to the plasma membrane. As shown in Fig. 1, each region was individually tested. Fused to the C-terminus of AQP3-tGFP, specific LHFPL5 regions were transiently expressed in HEK293 cells. The N-terminal domain LHFPL5^1–23^, ICL (LHFPL5^120–125^), ECL2 (LHFPL5^149–176^), and C-terminal domain LHFPL5^200–219^ did not prevent trafficking to the PM when transiently expressed as a part of the AGR system in HEK293 cells (Fig. 1A,C–E, white arrows). On the contrary, the LHFPL5 fragment ECL1, that encompasses amino acid residues 47–96 (LHFPL5^47–96^), which reside between the first and second transmembrane segment, does not allow any detectable plasma membrane (PM) localization (Fig. 1B), suggesting that the LHFPL5^47–96^ region precludes expression of LHFPL5 at the PM. Similar results were also obtained using CHO cells (Supplementary Fig. 1).

### E-Cadherin- and CD10-based specific reporters corroborate results obtained using AGR system

**Figure 2:**
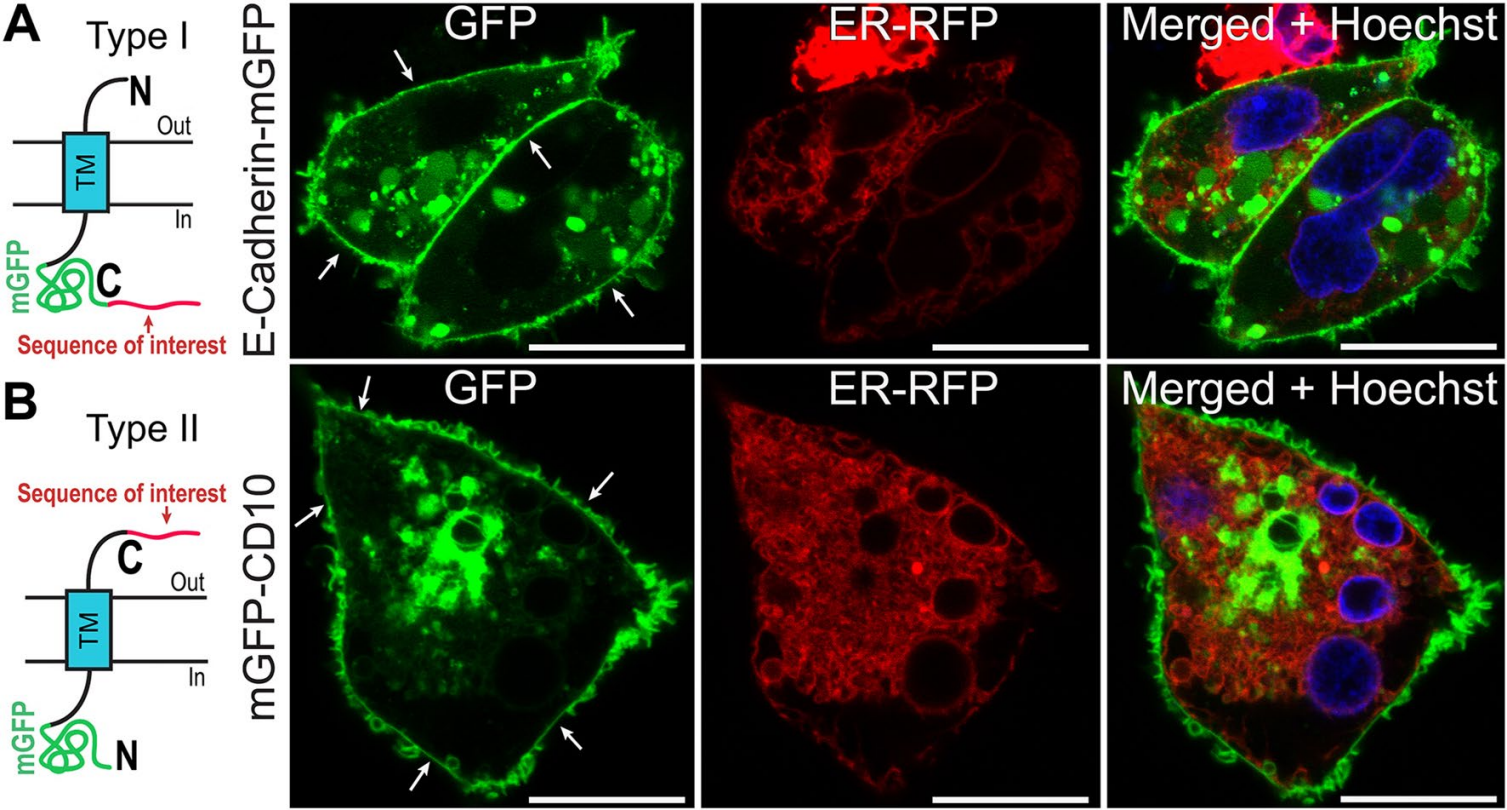
Employing E-Cadherin-mGFP- and mGFP-CD10-based reporters to test specific protein regions. (**A**) E-Cadherin belongs to the Type-I transmembrane family of proteins. E-Cadherin-mGFP (monomeric GFP) displays strong PM localization in transiently transfected HEK293 cells (white arrows), similar to the AGR system. E-Cadherin-mGFP reporter could be used to re-test intracellular loop domains and termini. (**B**) CD10 belongs to the family of Type-II transmembrane proteins. mGFP-CD10 shows intense PM localization in HEK293 cells. mGFP-CD10 reporter could be used to test extracellular loop domains and termini. Scale bars: 8 µm.

As a more specific alternative to the AGR system, we have designed a type I transmembrane^20^ protein-based E-Cadherin‐mGFP (monomeric GFP) plasma membrane reporter (Fig. 2A). E-Cadherin contains a single transmembrane segment (TM) and intracellular C‐terminal domain, and it displays intense PM staining when transiently expressed as a fusion protein with mGFP in HEK293 cells. We used the E-Cadherin‐mGFP reporter to examine potential endoplasmic reticulum retention regions in intracellular domains. CD10, a type II transmembrane protein^20^, also displays intense plasma membrane staining when tagged with mGFP, but possesses the opposite topology of E‐Cadherin and AQP3: CD10 contains a single TM segment and extracellular C‐terminal domain (Fig. 2B). We used mGFP‐CD10 to confirm whether extracellular domains contain any endoplasmic reticulum retention activity.

**Figure 3:**
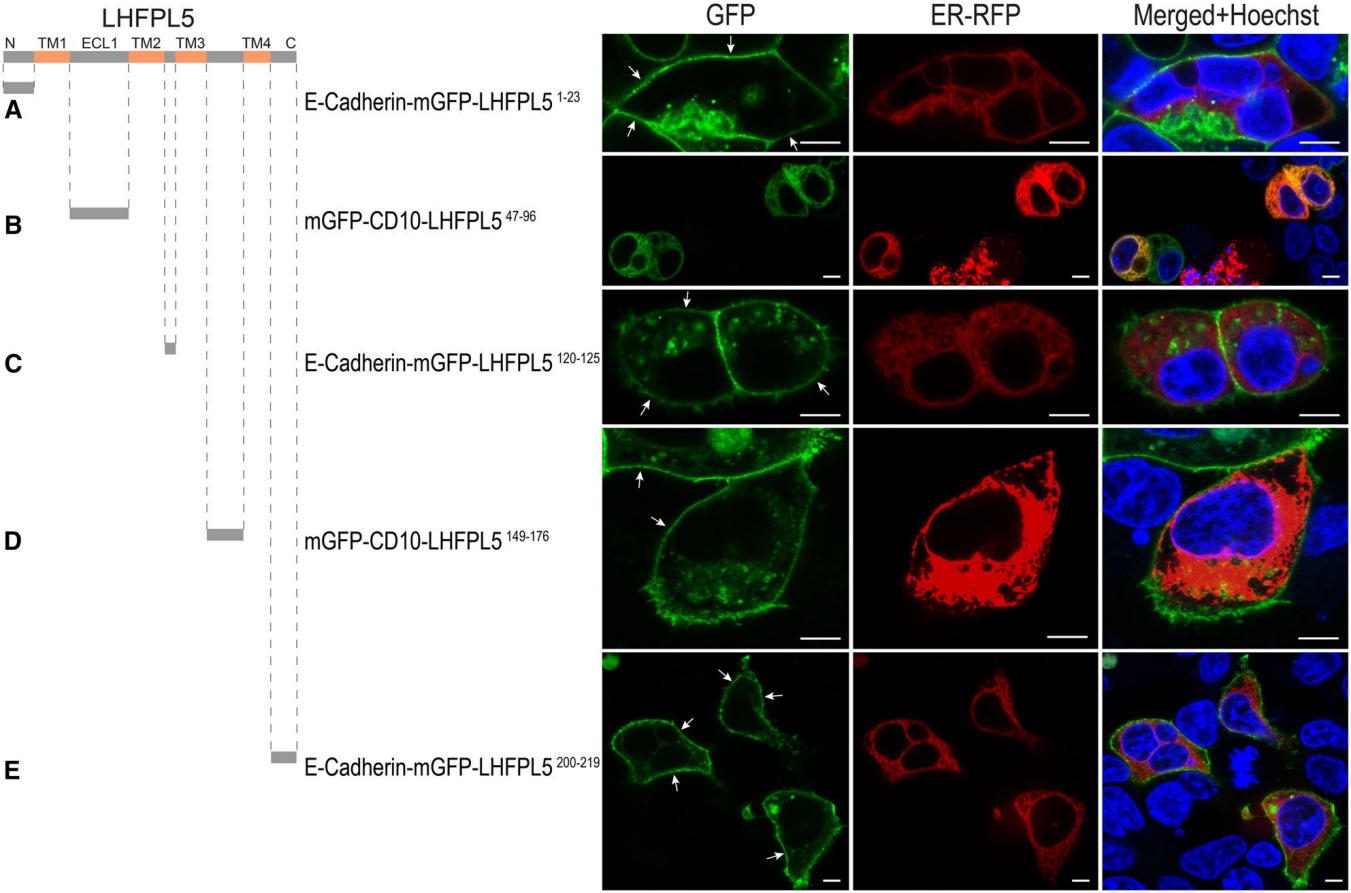
E-Cadherin- and CD10-based reporters confirm intracellular retention quality of the extracellular loop 1, LHFPL5^47–96^. (**A**) N-terminus of LHFPL5, residues LHFPL5^1–23^, when fused to the C-terminus of E-Cadherin-mGFP does not preclude reporter PM localization, confirming result obtained with AGR. (**B**) When extracellular loop 1 fragment LHFPL5^47–96^ was fused to C-terminus of mGFP-CD10 reporter, no detectable PM labeling was observed. These results confirm the data acquired using AGR, corroborating the ability of the LHFPL5^47–96^ to halt trafficking to the PM. (**C–E**) Intracellular loop 1 fragment LHFPL5^120–125^ (**C**), extracellular loop 2 fragment LHFPL5^149–176^ (**D**), and C-terminus fragment LHFPL5^200–219^ (**E**) did not prevent corresponding reporters E-Cadherin-mGFP-LHFPL5^120–125^ (**C**), mGFP-CD10-LHFPL5^149–176^ (**D**), and E-Cadherin-mGFP-LHFPL520^200–219^ (**E**) trafficking to the PM, confirming the result obtained by AGR. Scale bars: 10 µm.

We employed the E-Cadherin‐mGFP and mGFP‐CD10 reporters to confirm that retention of LHPL5^47–96^ (ECL1) was not due to the intracellular PM orientation imposed by the AGR system: the intracellular regions of LHFPL5 were fused to the C-terminus of E-Cahderin-mGFP, while the extracellular loops of LHFPL5 were attached to the C-terminus of mGFP-CD10 imitating their biological orientation within full length LHFPL5. As shown in Fig. 3, mGFP-CD10-LHPL5^47–96^ displayed predominantly ER retention while E-Cadherin-mGFP-LHFPL5^1–23^, E-Cadherin-mGFP-LHFPL5^120–125^, mGFP-CD10-LHFPL5^149–176^ and E-Cadherin-mGFP-LHFPL5^200–219^ displayed PM localization, in agreement with our AGR results (Fig. 1).

### Fragment sequences of LHFPL5^47–96^ (ECL1) do not block trafficking to the PM

**Figure 4:**
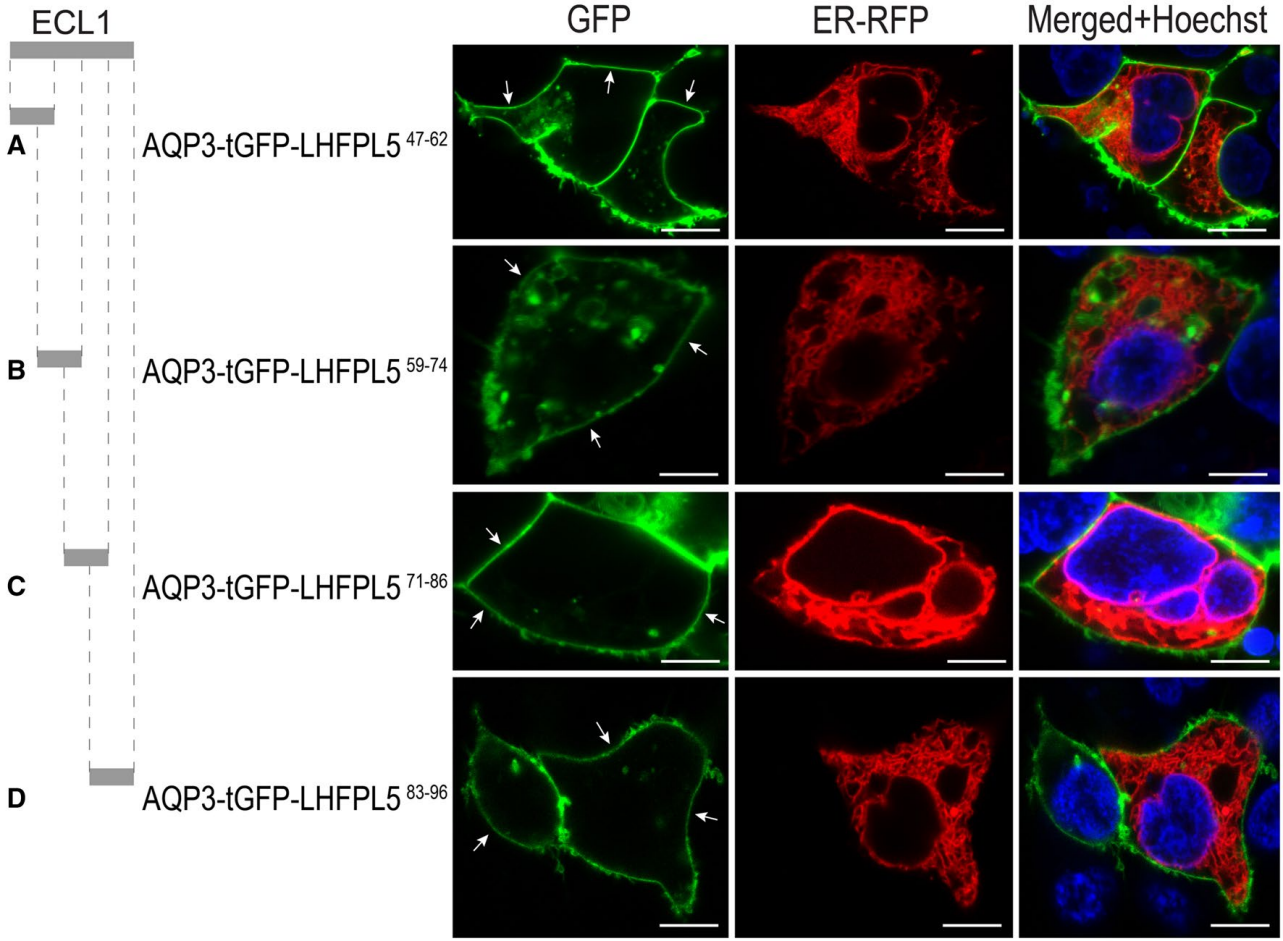
Further scanning ECL1 region LHFPL5^47–96^ to identify the exact fragment that prevents trafficking to the PM. (**A**) ECL1 fragment LHFPL5^47–62^ does not prevent reporter trafficking to the PM. When HEK293 cells were transfected with AQP3-tGFP-LHFPL5^47–62^, robust PM localization was observed (arrows). (**B**–**D**) Likewise, ECL1 fragments LHFPL5^59–74^ (**B**), LHFPL5^71–86^ (**C**), and (**D**) LHFPL5^83–96^ did not prevent PM localization (arrows). Scale bars: 8 µm.

Next, we tested whether the ECL1 of LHFPL5 contains an Omega-type signal sequence that abrogates detectable PM localization^18,19^. To narrow down the exact region of the potential signal, we split ECL1 of LHFPL5 encompassing amino acids LHFPL5^47–96^ into four overlapping fragments. To examine whether any ECL1 fragments prevent trafficking to the PM, fragments were fused to the C-terminus of the AGR system and expressed in HEK293. As shown in Fig. 4, we detected PM localization of LHFPL5^47–62^, LHFPL5^59–74^, LHFPL5^71–86^ or LHFPL5^83–96^, suggesting that most of the LHFPL5^47–96^ sequence is required for its ability to halt trafficking to the PM, or a small ER retention sequence was separated by the four fragment division.

### The transmembrane segments of LHFPL5 did not prevent trafficking to the PM

**Figure 5:**
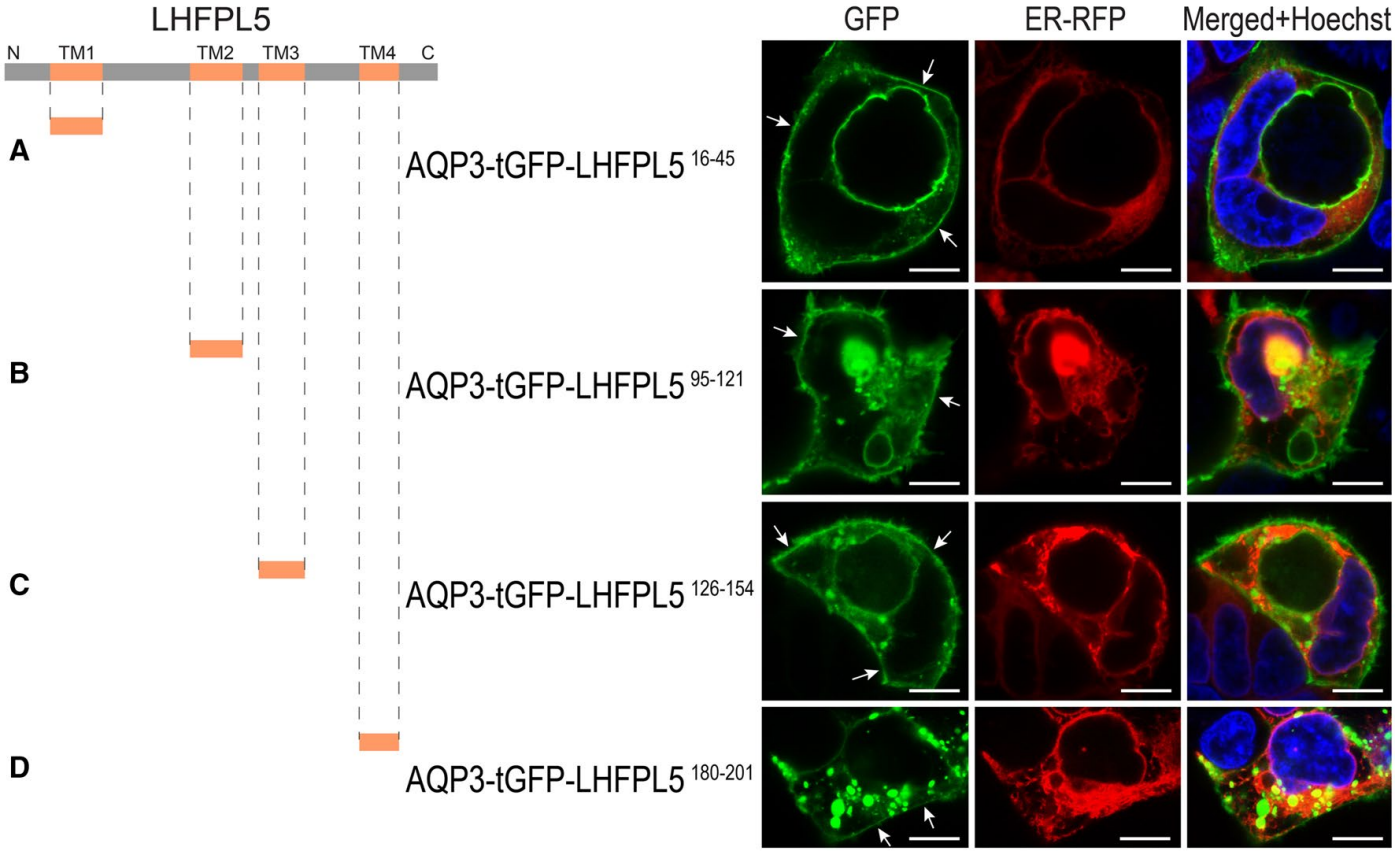
Assessing Transmembrane Segments of LHFPL5 for the ability to halt trafficking to the PM using AGR. (**A**) The first TM segment comprising residues of LHFPL5^16–45^ does not prevent reporter trafficking to the PM. PM localization was detected in HEK 293 cells transfected with AQP3-tGFP-LHFPL5^16–45^ (arrows). (**B–D**) Second TM segment amino acid sequence LHFPL5^95–121^ (**B**), third TM sequence LHFPL5^126–154^ (**C**) and fourth TM sequence LHFPL5^180–201^ (**D**) also did not prevent PM localization. Scale bars: 10 µm.

Transmembrane segments have been shown to participate in ER retention in certain proteins^21^, so we used the AGR system to determine whether any of the four TM segments of LHFPL5 contribute to ER retention of LHFPL5 when expressed heterologously. Using the same systematic approach described above, we C-terminally attached each TM sequence to AGR. The resulting constructs, AQP3-tGFP-LHFPL5^16–45^, AQP3-tGFP-LHFPL5^95–121^, AQP3-tGFP-LHFPL5^126–154^, or AQP3-tGFP-LHFPL5^180–201^ were transiently transfected in HEK293 cells. As shown in Fig. 5, PM localization was not prevented by any of the four TM sequences. These results suggest that none of the TMs of LHFPL5 contain a strong ER retention signal that hinders PM localization in heterologous cells.

## Discussion

Mutations in LHFPL5 may impair hair cell MET, leading to auditory and vestibular deficits in zebrafish, causing hearing loss and vestibular dysfunction in mice, and autosomal recessive nonsyndromic hearing loss in humans (DFN67)^5–8^. LHFPL5 has been shown to interact with PCDH15, another MET channel complex component, and fails to localize to the tips of stereocilia in the absence of PCDH15^3,10^. Moreover, LHFPL5 expressed in heterologous cells is retained intracellularly. The failure of LHFPL5 to traffic to the PM in a heterologous system is an obstacle to identifying and studying the function of this protein and its fundamental role in hearing. Here, using AGR and two additional novel reporters, E-Cadherin-mGFP and mGFP-CD10, we revealed the ability of the ECL1 region of LHFPL5 to halt trafficking to the PM in a heterologous cell line. When we examined the remaining LHFPL5 regions using the AGR system, including the intracellular and the second extracellular loops, both N- and C-termini, as well as the four TM segments, none prevented LHFPL5 localization to the PM. These results were confirmed with a couple of novel reporters, which are based on type I transmembrane protein E-Cadherin or type II transmembrane protein CD10. These novel reporters are more specific to the protein region of interest since they will express the LHFPL5 regions in their intrinsic cellular orientation. Our data also expose the value of the AGR method and suggest that more specific E-Cadherin and CD10 based novel reporters may be sufficient for future experiments. Since both AGR and CD10-based reporters lead to similar results, it is possible that the ER retaining machinery is present at both sides of the ER lumen to ensure the detection of a degron-like sequence.

We attempted to identify a shorter sequence within the ECL1 region of LHFPL5 that could be responsible for preventing trafficking to the PM by examining four overlapping ECL1 fragments. None of the fragments retained the ability to prevent reporter trafficking to the PM; all AGR reporter containing fragments of ECL1 sequence reached the PM when expressed in HEK293 cells, suggesting that none of the fragments of the ECL1 region tested contained an intact ER retention sequence that could halt trafficking to the PM. Furthermore, each TM segment of LHFPL5 was assessed for their potential role in intracellular retention. However, all of the AGR construct containing each TM segment trafficked to the PM, suggesting a minor role for these protein regions in LHFPL5 intracellular retention. Thus, using the AGR system, we found that the ECL1 region LHFPL5^47–96^ was the only LHFPL5 fragment that completely abrogated any detectable PM localization. Interestingly, the ECL1 contains the region known to interact with PCDH15^12^. Thus, our results further suggest that the presence of cell-specific protein partners may mask ER-retention regions and facilitate trafficking to the PM in stereocilia^19^.

Except for a small deletion resulting in a frameshift (p.31 fs)^22^, known deafness mutations in LHFPL5 do not localize to interaction sites of LHFPL5 and PCDH15^12^, suggesting that these mutations do not alter the LHFPL5-PCDH15 interaction. As LHFPL5 Y127C, C161F, T165M and R176L deafness-causing mutants interact with PCDH15^3^, it is expected that LHFPL5 interacts with components of the MET complex at different sites as well^3,12,15^.

LHFPL5 structure resembles that of claudin proteins, sharing common features. The two spaced apart cysteine residues in ECL1, which are conserved in the claudin proteins, are also present in LHFPL5. Additionally, LHFPL5 contains the claudin signature sequence Glycine-Leucine-Tryptophan (GLW) within the ECL1. Mutations of these cysteine residues abolish ligand binding and the ability of claudins to form an ion barrier, suggesting an essential role in the correct folding of the ECL1^23,24^. Furthermore, the tryptophan and arginine residues located at the beginning and end of the ECL1 are conserved in claudins and hypothesized to have an essential role in protein folding^25^. This putative sequence is believed to be essential for the correct folding of loop 1, ligand recognition, and trans interaction between two claudins expressed in various cell types^25–27^.

Removing or replacing the whole ECL1 region of the LHFPL5 may allow for PM localization in heterologous cells, although misfolding and degrading should not be ruled out. Future studies should be aimed at finding the exact sequence of the omega-type signal within ECL1 responsible for the intracellular retention. Once identified, deletion of the omega-type signal or, alternatively, alanine and/or serine substitution mutagenesis could be performed to rescue LHFPL5 trafficking to the PM.

## Materials and methods

### Plasmid constructs

The AQP3-tGFP-pcDNA5/FRT plasmid, which is abbreviated here as AQP3-tGFP and termed as the AGR system, was synthesized as previously described^18,19^. *Lhfp5* sequences based on *Lhfp5* (NP_872354) were subcloned into the AQP3-GFP-pcDNA5/FRT plasmid using Hpa1 and EcoRV restriction sites, synthesized by Genscript (Piscatawas, NJ), and confirmed by sequencing.

### Cell culture, transfection

HEK293 cells were purchased from Life Sciences (Carlsbad, CA) and seeded in 12-well glass-bottom plates (Cellvis, CA), cultured in DMEM/F12 media (Life Technologies, CA) supplemented with 10% Fetal Bovine Serum and 1% pen/strep. Cells were transfected as previously described^18,19^. Briefly, on the day of transfection, spent media was replaced by fresh media and HEK293 cells were transfected with plasmid constructs using ExpiFectamine 293 (Life Technologies, CA). Red Fluorescent Protein (RFP) based BacMam 2.0 constructs, specific for ER, were co-transfected the same day following manufacturer’s instructions (ThermoFisher Scientific, MA). This ER-RFP marker was used to properly identify the ER membrane, which sometimes can be easily confused for PM. To label the cell nucleus, Hoechst staining was used (Life Technologies, CA). Cells were examined with an SP8 confocal fluorescence microscope using 63 × 1.4 NA objective, Leica (Wetzlar, Germany). We captured 3 to 6 fields of view per dish, using two dishes per construct in every experiment. All experiments were performed in duplicates on different days to ensure reproducibility.

## Supplementary Information

Supplementary Figure 1.

## Data Availability

The datasets used and/or analyzed during the current study available from the corresponding author on reasonable request.
