# Supplementary material for: Multiple plasma membrane reporters discern LHFPL5 region that blocks trafficking to the plasma membrane
Source: Sci Rep. 2023 Feb 13;13:2528. doi: 10.1038/s41598-023-28045-w (PMC9925724; doi:10.1038/s41598-023-28045-w)
Supplement: Supplementary file 1 — Supplementary Figure 1. [file 41598_2023_28045_MOESM1_ESM.pdf]

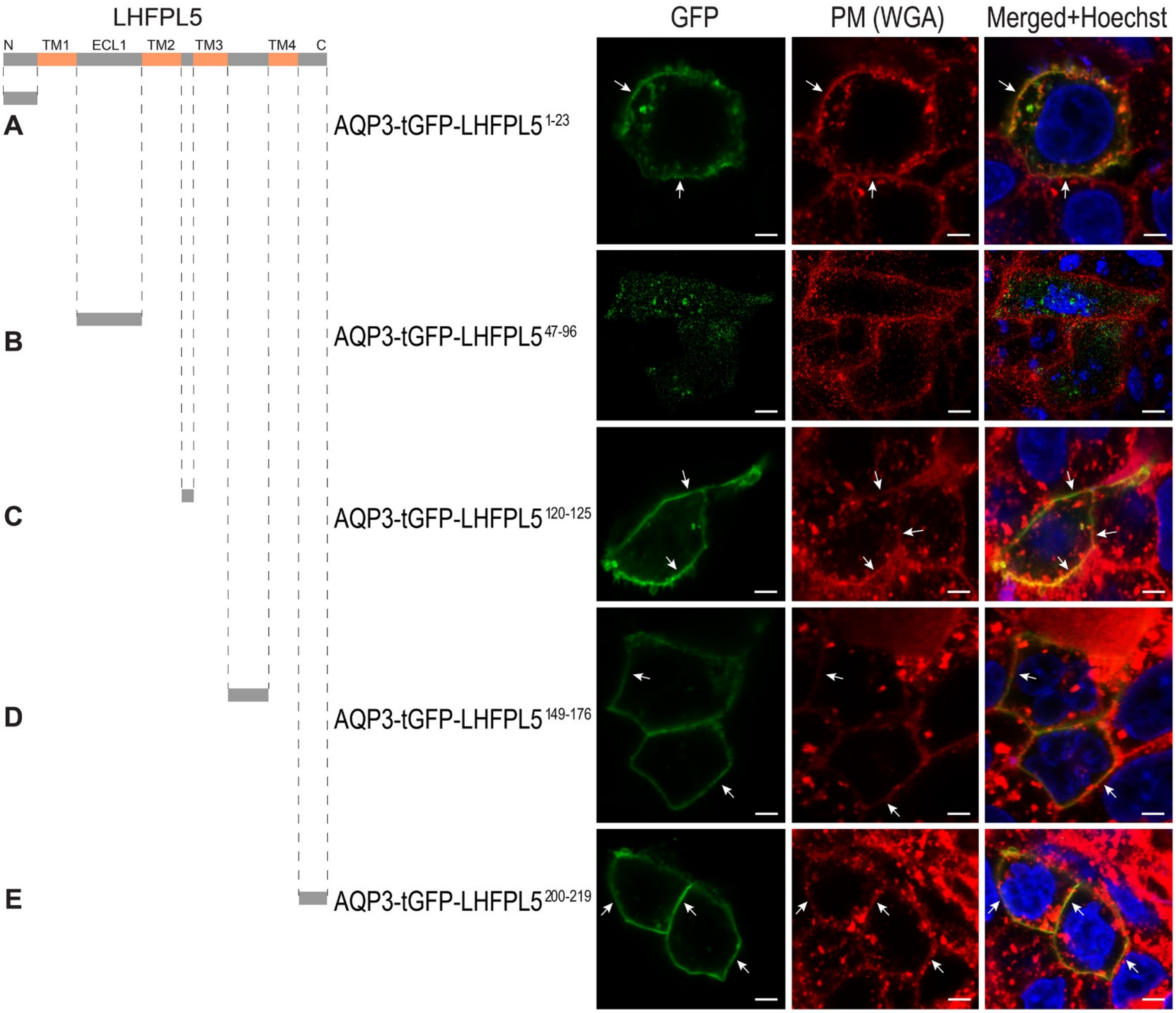

Supplement Figure 1. Key experiments are reproduced in CHO cell lines. **A**) The N-terminus fragment of LHFPL5 comprising residues LHFPL5$^{1\text{-}23}$, does not preclude trafficking of the AGR system to the plasma membrane (PM) when expressed in CHO cells. **B**) Extracellular loop 1, consisting of the LHFPL5$^{47\text{-}96}$ fragment, also halts any detectable trafficking to the PM when expressed in CHO cells. **C-E**) AGR-based constructs containing the remaining intracellular loop 1, LHFPL5$^{120\text{-}125}$ (C), extracellular loop 2, LHFPL5$^{149\text{-}176}$ (D) or the C-terminus, LHFPL5$^{200\text{-}219}$ (E) show PM labeling when expressed in CHO cells. To label the PM, wheat germ agglutinin (WGA) Alexa Fluor 594 conjugate was used (ThermoFisher Scientific, MA). Scale bars: 10 μm.
